# Readmissions after cytoreductive surgery and hyperthermic intraperitoneal chemotherapy—a national population-based study

**DOI:** 10.1186/s12957-020-01837-4

**Published:** 2020-04-06

**Authors:** Paul Dranichnikov, Wilhelm Graf, Peter H. Cashin

**Affiliations:** Department of Surgical Sciences, Colorectal Surgery Section, Uppsala University Hospital, 1st Floor, Entrance 70, S-751 85 Uppsala, Sweden

**Keywords:** Readmission, Peritoneal metastases, HIPEC, Morbidity, Gastric resection

## Abstract

**Background:**

Comprehensive readmission morbidity studies after cytoreductive surgery (CRS) and hyperthermic intraperitoneal chemotherapy (HIPEC) are scarce. This study aimed to investigate readmissions and in-hospital morbidity after CRS and HIPEC.

**Methods:**

The national in-hospital patient register was used to identify patients via the HIPEC ICD code JAQ10 2004–2014. Data were retrieved from the index CRS/HIPEC treatment and from all HIPEC-related readmissions within 6 months. Univariate/multivariate logistical analyses were performed to identify risk factors for reinterventions and readmissions.

**Results:**

A total of 519 patients (mean age 56 years) had a mean hospital stay of 27 days. Within 6 months, 150 readmissions for adverse events were observed in 129 patients (25%) with 67 patients requiring an intervention (13%). Totally 179 patients (34%) required a reintervention during the first 6 months with 85 (16%) requiring a reoperation. Of these 179 patients, 83 patients (46%) did not undergo the intervention at the HIPEC centre. Gastric resection was the only independent risk factor for in-hospital intervention, and advanced age for readmission.

**Conclusion:**

Morbidity causing HIPEC-related readmission was higher than expected with almost half of the interventions occurring outside the HIPEC centre. Gastric resection and high age are independent predictors of morbidity and readmission.

## Background

Cytoreductive surgery (CRS) combined with hyperthermic intraperitoneal chemotherapy (HIPEC) is an accepted treatment for peritoneal metastases (PM) of appendiceal and colorectal origin, peritoneal mesothelioma and selected cases of advanced ovarian cancer [1–7]. During the past decade, several randomized trials within the area of PM treatment have shown promising results for ovarian, colorectal and even gastric cancers [8–12]. Patients undergoing CRS and HIPEC for peritoneal surface malignancy are at high risk for a wide range of morbidity. The most common forms of morbidity are postoperative infections, haemorrhage complications, enterocutaneous fistula and haematological toxicity. Chua et al. suggest that morbidity after CRS and HIPEC is similar to that of other major gastrointestinal surgeries, such as a Whipple procedure and oesophageal resection [13–17]. Likewise, CRS is complex and consists of multiple procedures, including a series of peritonectomy and visceral resections that are performed in order to visibly clear the abdominal cavity and pelvis of malignant nodules [18–22]. Morbidity in the form of surgical complications is common and has a significant impact on the quality of life. The proportion of patients experiencing postoperative morbidity has been close to 50%, and reoperation rates have ranged from 11 to 28% [23–28]. Some complications present before hospital discharge, whilst others occur within 6 months. Despite this, the frequency of and reasons for readmissions after CRS and HIPEC have been poorly investigated [14]. The primary objective of this national register study was to analyse the incidence of readmissions after CRS and HIPEC in Sweden. A secondary objective was to assess the national overall morbidity rates and risk factors for morbidity requiring readmission or intervention.

## Materials and methods

Data were retrieved from Sweden’s National Patient Register and the Cause of Death Register and included all patients with a Swedish social security number who underwent their first HIPEC procedure in Sweden. This was done by using the HIPEC ICD code JAQ10 from January 1, 2004, until June 30, 2014, for HIPEC performed in all four HIPEC centres in Sweden. As the code JAQ10 was not used consistently early on, when there was only one centre in Sweden (Uppsala University Hospital), the cohort from the National Patient Register was combined with the HIPEC register at Uppsala in order to have a complete cohort. All patients who underwent HIPEC procedure and had at least 6 months of follow-up after index HIPEC were included in the cohort regardless of surgical result. All hospital admissions were retrieved from the first HIPEC treatment/index HIPEC (some patients were treated several times) until 6 months postoperatively. No patients included in this cohort underwent second HIPEC within 6 months after index HIPEC. The reason for excluding repeat HIPEC procedures is that the indication and selection for a repeat HIPEC procedure are different from the index procedures (i.e. usually more limited tumour extension) making them difficult to compare. Study observation ended on December 31, 2014. The following information was registered from each hospital admission: age, gender, primary tumour site, coded surgical procedures, coded postoperative morbidity diagnoses, all reoperations or interventional coded therapies, the date for index surgical procedures and the date for reoperation and readmissions required for reinterventions. Since the study design is based on register data extracted using ICD codes, the authors have not been able to perform adjustments to the comorbidity nor have the authors been able to include intraoperative data such as operation duration, perioperative bleeding, detailed perioperative injuries and HIPEC regimens.

The Swedish Cause of Death Register was used to ascertain the date and cause of death in the cohort. Interventions were categorized into radiological, endoscopic or surgical interventions. Hospital stays related to early recurrence, other anti-tumour treatment (or complications thereof) or hospice care were not considered.

The study was approved by the regional ethics committee for the Uppsala region, Sweden (reference no. 2015/367).

### Statistics

Statistical analysis was performed using *Statistica 64* software for Windows (Version 13.3, Dell Software, Round Rock, TX, USA). Descriptive statistics included mean, median, percentage and range. Univariate logistical analyses were performed on age, gender and operative procedures to identify potential risk factors for the three endpoints—in-hospital intervention, HIPEC-related readmission and readmission requiring an intervention. All variables with a statistically significant correlation to these endpoints in univariate analyses were tested in multivariate logistical regression analysis to identify independent risk factors for the mentioned endpoints. Logistical regression results were presented as odds ratio and 95% confidence interval. Statistical significance was defined at *p* < 0.05.

## Results

### Demographic and clinical overview

In total, 519 patients were included: 222 males (43%) and 297 females (57%) with a mean age of 56 years (range 13–78). The most common primary tumour site was the appendix (*n* = 235) including all subtypes of appendiceal neoplasms. The mean number of organ resections was 4 (range 0–11). Common organ resections were colon (*n* = 365, 70%), parietal peritoneum (*n* = 484, 93%) and larger omentum (*n* = 452, 87%, Table 1).

**Table 1:** Demographics of all 519 patients who underwent CRS + HIPEC in Sweden 2004–2014

Clinical values	Results, *n*	Percentage
Age (mean)	56 [range 13–78]	
Gender (male to female)	222:297	43:57
Primary tumour		
Colorectal	168	32
Appendix (including PMP/DPAM/PMCA)	235	45
Gastric	9	2
Small intestine	15	3
Gynaecological	25	5
Mesothelioma	24	5
Unspecified	43	8
Referral centre		
Patient from region with HIPEC centre	62	12
Patient from region with no HIPEC centre	315	61
Patient from unspecified referral region	142	27
Operation parameters		
Total stomas	225	43
Colostomy	64	28.4
Ileostomy	161	71.5
Splenectomy	181	34.8
Hysterectomy	114	38 (of 297 females)
Salpingo-oophorectomy	162	54.5 (of 297 females)
Resection of vagina	15	5 (of 297 females)
Orchidectomy	3	1 (of 222 males)
Resection of the seminal vesicle	11	5 (of 222 males)
Gastric resection	36	7
Hepatic resection	95	18
Pancreatic resection	10	2
Cholecystectomy	154	30
Small bowel resection	232	45
Appendectomy	41	8
Any colonic resection	365	70.3
Rectal resection	193	37
Resection of the bladder	15	3
Resection of the ureter	15	3
Repair of abdominal hernia	13	2.5
Survival and mortality		
Alive at study end December 31, 2014	314	60.5
In-hospital mortality	5	0.96
Dead within 6 months	17	3.2

### Six-month readmission rate and risk analysis

**Fig. 1:**
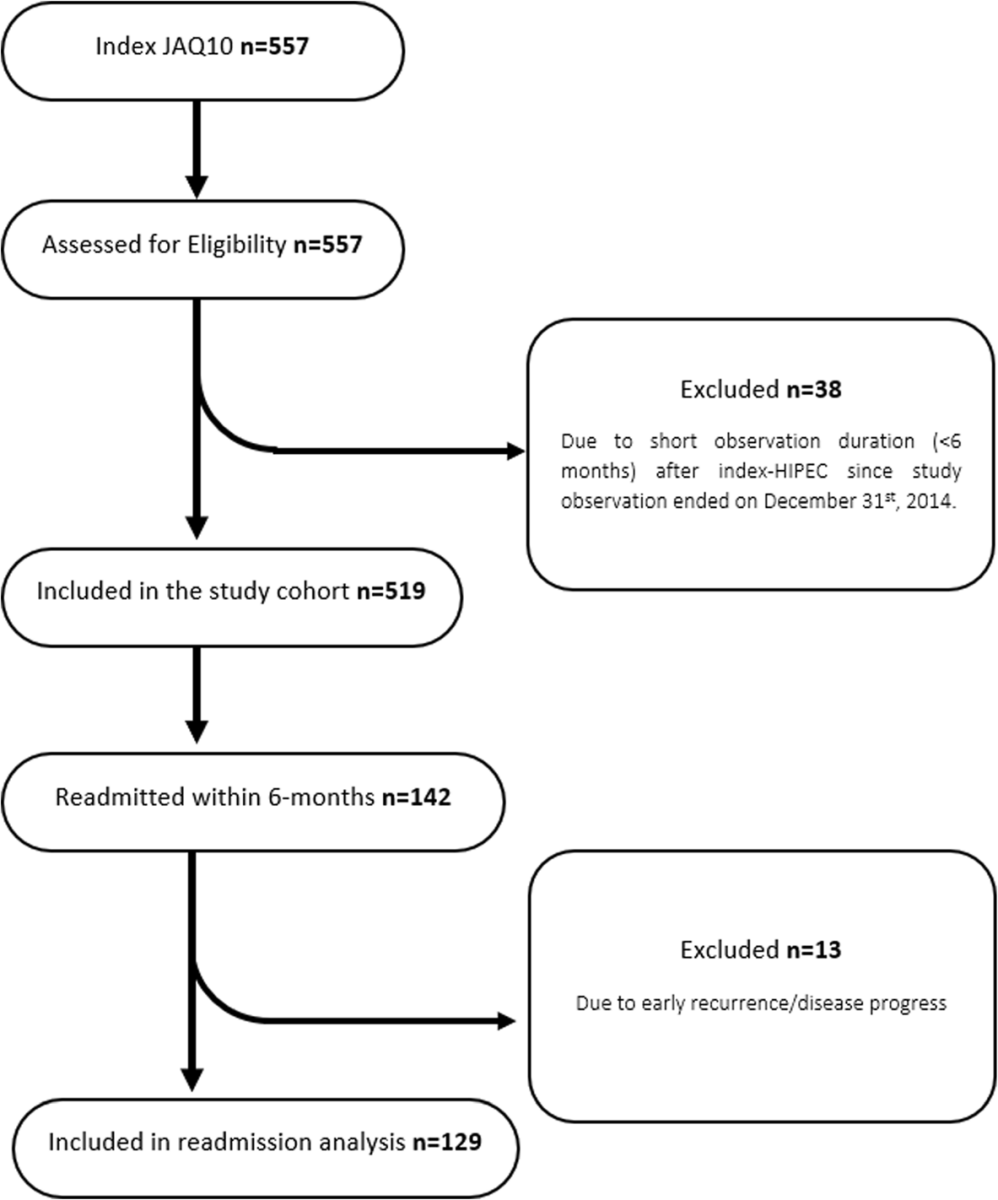
Flowchart of study inclusion and readmission analysis

One hundred and forty-two patients were readmitted within 6 months (27%). However, 13 patients (2.5%) were excluded due to disease progress-related readmission that resulted in HIPEC-related readmission group of 129 patients (Fig. 1). In total, 150 HIPEC-related readmissions occurred in those 129 patients (25%), with 83 interventions performed on 67 of them (i.e. 13% of the entire cohort required a readmission intervention within 6 months, Tables 2 and 3). Complications at readmission fell into three categories: gastrointestinal (*n* = 95), cardiovascular (*n* = 25) and miscellaneous (*n* = 30).

**Table 2:** In-hospital and readmission adverse events and interventions of the cohort (*n* = 519)

Adverse events	*n*	Interventions	*n*
Patients with any in-hospital adverse event	261	Patients with any in-hospital intervention	112
At HIPEC hospital only	221	At HIPEC hospital only	96
At both hospitals	17	At both hospitals	4
At referral hospital only	23	At referral hospital only	12
Patients with any HIPEC-related readmission adverse event	129	Patients with any readmission intervention	67
Total number of patients with any HIPEC-related adverse event within 6 months	319	Total number of patients with any HIPEC-related intervention within 6 months	158

**Table 3:** Details of readmission adverse events and interventions within 6 months

Adverse events in 129 patients	*n* = 150	Interventions in 67 patients	*n* = 83
Cardiovascular events	**25**	Radiological interventions	**18**
Pulmonary embolism	11	Thoracentesis	4
Venous thrombosis	7	Abdominal drainage	10
Arterial thrombosis	2	Nephrostomy	4
Other	5	Endoscopic interventions	**12**
Gastrointestinal events	**95**	Gastroscopy	10
Abdominal infection	25	Rectoscopy	1
Anastomotic insufficiency	2	Cystoscopy	1
Stoma complication	4	Surgical interventions	**53**
Bowel obstruction	49	Bowel obstruction	13
Fistula	10	Wound dehiscence	1
Gastrointestinal bleeding	5	Anastomotic insufficiency	2
Miscellaneous	**30**	Abdominal infection	8
Infection	13	Gastrointestinal bleeding	1
Pulmonary	4	Fistula	6
Neutropaenia	4	Stoma reversal	13
Renal failure	6	Minor surgical procedure	9
Other/unspecified	3		

Age at treatment (OR 1.02, CI 1.00–1.03, *p* = 0.004) and any colonic resection (OD 1.85, CI 1.03–3.31, *p* = 0.03) were associated with a significantly higher risk for a HIPEC-related readmission. The risk for readmission requiring an intervention was significantly increased by advanced age at treatment (OR 1.02, CI 1.00–1.04, *p* = 0.02). Late HIPEC-related complications were noted with 48.7% of readmissions occurring between 90 days and 6 months.

### Summary of 6-month interventions and mortality

An intervention during the first postoperative 6 months (both postoperative in-hospital and at readmission) was required in 34% of the patients (*n* = 179) including 16% requiring a surgical reoperation (*n* = 85). Out of these 179 patients requiring an intervention, 83 patients (46%) received it at the referral hospital or during a readmission.

In-hospital mortality was noted in five patients (1%). One patient died on day 73 due to abdominal sepsis caused by an enterocutaneous fistula. Another patient died on day 25 due to cerebral infarction.

Two patients died on days 54 and 190 due to circulatory failure caused by haemorrhage. Finally, one patient died on day 67 due to deep infection caused by anastomosis insufficiency.

### In-hospital morbidity and risk analysis

A total of 438 in-hospital adverse events occurred in 261 patients (50%): 221 patients at the HIPEC centre, 23 patients at the referral hospital and 17 patients at both hospitals. One hundred and forty-five in-hospital interventions occurred in 112 patients: 96 patients at the HIPEC centre, 12 patients at the referral hospital and four patients at both hospitals (Table 2).

All adverse events and corresponding interventions are summarized in Table 4. The mean number of days to an in-hospital surgical intervention (return to operating theatre) was 11.9 (0–51).

**Table 4:** Details of in-hospital adverse events and interventions

In-hospital adverse events in 261 patients	*n* = 438	In-hospital interventions in 112 patients	*n* = 145
Cardiovascular events	**40**	Radiological interventions	**61**
Heart infarction	2	Thoracentesis	33
Cerebral vascular lesion	2	Abdominal drainage	23
Atrial fibrillation	20	Nephrostomy	3
Deep vein thrombosis	4	PTC	2
Pulmonary embolism	7	Endoscopic interventions	**11**
Cardiac arrest	2	Gastroscopy	5
Cardiomyopathy	1	ERCP	3
Other, unspecified	2	Procto/rectoscopy	2
Respiratory events	**93**	Bronchoscopy	1
Pleural effusion	52	Surgical interventions	**76**
Pneumonia	22	Wound dehiscence	9
Haemopneumothorax	10	Anastomotic insufficiency	9
Acute respiratory failure	9	Exploratory laparotomy	8
Gastrointestinal events	**128**	Haemorrhage/haematoma	5
Abdominal infection	51	Bowel obstruction	4
Anastomotic insufficiency	13	Enterorrhaphy	4
Bowel obstruction	20	Resection of the small intestine	3
Wound dehiscence	16	Bile leakage	3
Intra-abdominal bleeding	14	Gastrorrhaphy	3
Fistula	4	Abdominal sepsis	2
Gastrointestinal perforation	7	Splenectomy	1
Perforation of the bile duct	3	Cholecystectomy	1
Urological events	**52**	Other/unspecified	24
Acute renal failure	26	Miscellaneous	**3**
Urinary tract infection	19	Dialysis	3
Hydronephrosis	6		
Renal infarction	1		
Miscellaneous	**103**		
Neutropaenia	68		
Isolation	10		
Other infections	35		
Perioperative injuries	**22**		

Cardiovascular complications represented 10% of all in-hospital morbidity. Two cardiac arrests with successful resuscitation and a single case of cardiomyopathy due to chemotherapy treatment were reported. Respiratory complications represented 21% of all in-hospital morbidity (*n* = 93) whilst urological complications comprised 12% (*n* = 52).

The majority of urological adverse events involved acute renal failure: 47% (*n* = 26) with three renal failures requiring dialysis treatment. Furthermore, one case of minor renal infarction was reported. All neutropaenia cases (*n* = 68) were treated with Neupogen® (AMGEN Inc., Thousand Oaks, CA, USA) (see Table 4).

The mean total in-hospital stay, including HIPEC centre and referral hospital postoperative care, was 27 days (range 7–190) with a mean length of stay at the HIPEC centre of 19 days and 8 days at the referral hospital.

Univariate logistical analysis showed that gastric resection, splenectomy and number of resections had a significant *p* value for risk for in-hospital intervention (Table 5). However, gastric resection was the only independent risk factor for in-hospital intervention in the multivariate logistical regression analysis (*p* = 0.02, Table 6).

**Table 5:** Univariate logistical regression using the three endpoints of the study

Characteristics	Risk for in-hospital intervention	*p* value	HIPEC-related readmission	*p* value	Readmission requiring intervention	*p* value
Age at treatment	0.98 (0.97–1.00)	0.13	1.02 (1.00–1.013	0.004	1.02 (1.00–1.04)	0.01
Gender						
Female	Ref		Ref		Ref	
Male	1.07 (0.70–1.64)	0.74	1.07 (0.72–1.61)	0.70	1.29 (0.76–2.20)	0.3
Gastric resection (36)	2.87 (1.43–5.79)	0.003	1.17 (0.55–2.51)	0.67	1.38 (0.55–3.46)	0.48
Pancreatectomy (10)	2.50 (0.69–9.03)	0.16	2.04 (0.56–7.37)	0.27	2.97 (0.75–11.81)	0.12
Liver resection (95)	1.05 (0.61–1.80)	0.85	0.89 (0.52–1.50)	0.67	0.56 (0.26–1.23)	0.15
Cholecystectomy (154)	1.24 (0.79–1.94)	0.34	1.66 (1.09–2.53)	0.01	1.60 (0.94–2.73)	0.08
Splenectomy (181)	1.81 (1.18–2.78)	0.006	1.42 (0.94–2.14)	0.08	1.12 (0.66–1.92)	0.65
Small bowel resection (232)	1.16 (0.76–1.78)	0.46	1.47 (0.98–2.19)	0.05	1.74 (1.03–2.92)	0.03
Any colonic resection (365)	1.24 (0.77–2.00)	0.35	2.48 (1.50–4.12)	0.0003	2.3 (1.19–4.63)	0.01
Appendectomy (41)	0.88 (0.39–1.96)	0.76	0.83 (0.38–1.80)	0.65	0.32 (0.07–1.38)	0.12
Rectal resection (193)	1.38 (0.90–2.11)	0.13	1.90 (1.27–2.86)	0.001	1.77 (1.05–2.96)	0.02
Ureter resection (15)	0.25 (0.03–1.96)	0.19	2.06 (0.72–5.91)	0.17	3.56 (1.17–10.77)	0.02
Bladder resection (15)	0.25 (0.03–1.96)	0.19	2.73 (0.97–7.71)	0.05	2.54 (0.78–8.23)	0.11
Abdominal hernia repair (13)	2.35 (0.75–7.35)	0.13	1.35 (0.41–4.47)	0.61	1.23 (0.26–5.69)	0.78
Number of resections	1.11 (1.01–1.22)	0.02	1.20 (1.09–1.31)	0.00007	1.21 (1.08–1.36)	0.0007

Confidence interval between parentheses; *N/A* not applicable

**Table 6:** Multivariate logistical analyses according to three endpoints

Characteristics	Risk for in-hospital intervention	*p* value	HIPEC-related readmission	*p* value	Readmission requiring intervention	*p* value
Age at treatment	N/A	N/A	1.02 (1.00–1.03)	0.004	1.02 (1.00–1.04)	0.02
Gastric resection(36)	2.34 (1.13–4.87)	0.02	N/A	N/A	N/A	N/A
Splenectomy (181)	1.53 (0.90–2.59)	0.10	N/A	N/A	N/A	N/A
Small bowel resection (232)	N/A	N/A	1.09 (0.69–1.71)	0.69	1.26 (0.71–2.22)	0.41
Cholecystectomy (154)	N/A	N/A	1.16 (0.66–2.03)	0.58	N/A	N/A
Any colonic resection (365)	N/A	N/A	1.85 (1.03–3.31)	0.03	1.49 (0.68–3.26)	0.30
Rectal resection (193)	N/A	N/A	1.37 (0.82–2.26)	0.21	1.11 (0.59–2.11)	0.73
Ureter resection (15)	N/A	N/A	N/A	N/A	2.24 (0.70–7.11)	0.16
Number of resections	0.9 (0.86–1.09)	0.65	0.94 (0.80–1.10)	0.45	0.89 (0.76–1.04)	0.15

Confidence interval between parentheses; *N/A* not applicable

### Overall survival

At the end of the study observation date of December 31, 2014, 61% of the cohort was still alive.

## Discussion

This is the first national, comprehensive readmission and morbidity study within the field of CRS and HIPEC. Furthermore, this study is the first in the field to investigate morbidity up to 6 months after first discharge after CRS and HIPEC. Due to the Swedish registry system, we were able to consistently retrieve all hospital admission and discharge diagnosis codes and interventional coding data within 6 months of CRS/HIPEC surgery.

Several morbidity studies have been published including a systematic review, but few studies have looked at the readmission rate after CRS and HIPEC [14, 24]. Our study examined the full extent of readmission and morbidity, not only from the HIPEC centre but also from the referral hospital where many of the patients receive postoperative care before being discharged from the hospital. Readmission data were captured regardless of where in Sweden the patient was admitted, since the national in-hospital patient register automatically collects ICD-coded discharge information from all Swedish hospitals. As such, operating codes, and medical diagnosis codes for both diseases and complications, are available. Nevertheless, grading is not possible through this register without acquiring all patients’ individual medical records. However, since all interventions are coded (including both surgical and radiological procedures), there is a good correspondence between the Clavien-Dindo grades III–IV adverse events and the patients’ interventional codes.

The most important finding in this study was the number of interventions occurring in the referral hospital or during a HIPEC-related readmission. In total, 46% of all patients (83/179, Table 3) requiring an intervention in the first 6 months did not receive it during the postoperative stay at the HIPEC centre but at the referring hospital or during a readmission. As such, most morbidity studies underestimate the true morbidity and reoperation rate unless data from the referral hospital are considered.

Improved communication between referring hospitals and HIPEC centres is needed if we are to properly assess and manage patients with complications after CRS and HIPEC surgery. Furthermore, it is essential for patients suffering from peritoneal surface malignancy to be aware of and understand the possible long-term risks following this procedure.

The postoperative in-hospital intervention rate of 27% with a slight increase to 34% within 6 months is comparable to that seen in previous studies (grades III/IV morbidity up to 52%) [13, 25]. Likewise, the need for surgical intervention within the first 6 months (including both postoperative in-hospital and readmission) was 16%, which is also comparable to previous studies (11–26.8%) [26–28].

Readmissions, morbidity and mortality after CRS and HIPEC seem to differ from other abdominal surgical procedures. Some of the reasons are identified in this study. Every CRS and HIPEC treatment is individually adapted regarding different resections and reconstruction approaches, depending on the extent of peritoneal surface malignancy and the characteristics of the patient: the more extensive the resection on a frail patient, the higher the risk for adverse events and readmission. Pancreaticoduodenectomy (considered to be the closest to HIPEC regarding complexity of procedure) has a risk of up to 21% for early hospital readmission within 30 days [16]. Ahmad et al. reported 15% readmission within 30 days and 19% within 90 days after the same procedure [15], whilst Bagante et al. reported a 23% readmission rate within 90 days after hepato-pancreatic surgery for malignant disease [29]. Our study had a very similar early readmission rate within 30 days of 14%, although it increased to 25% after 6 months. The time frame for studying postoperative readmissions and morbidity in this study has been expanded to 6 months in order to investigate possible late HIPEC-related readmission since no other study in the field had done that before.

Furthermore, the readmission rate after 90 days and within 6 months postoperatively in this study was 48.7% of the total number of readmissions.

The number of surgical resections performed was univariately significant for all three endpoints in the logistical analyses. However, it lost its significance in all three multivariate analyses. This is probably due to correlation with the other resection variables. This is however interesting as it seems that the overall number of resections is not the issue but rather certain specific resections that are more problematic.

A gastric resection was an independent risk factor for in-hospital intervention. A systematic review of survival and morbidity in gastric cancer patients with peritoneal surface malignancy undergoing CRS and HIPEC done by Gill et al. reported an overall morbidity of 21.5%. Furthermore, the most commonly reported complications were abscess, fistula and anastomotic leak [30].

The most common complications in our cohort of patients who had gastric resection (*n* = 36) were abscess, anastomotic leak and wound dehiscence (*n* = 15). The need for gastric resection should be evaluated in relation to the overall risk of other complications. It may increase the risk of in-hospital complications requiring an intervention threefold.

The multivariate analyses for HIPEC-related readmission showed that any colonic resection was a significant risk factor (*p* = 0.03) whilst the same analysis for HIPEC-related readmission and readmission requiring an intervention both showed that age was the only independent significant risk factor (*p* = 0.004 and *p* = 0.02, respectively, Table 6). Few studies have investigated morbidity after CRS and HIPEC in relation to age at treatment [31–33].

Elias et al. reported no correlation between age and occurrence of intra-abdominal complications, whilst Beckert et al. reported that CRS and HIPEC are not associated with either grades III–IV morbidity or surgery-related mortality in elderly patients [31, 33].

There may be a need to further explore this aspect, considering the increased risk for both HIPEC-related readmission and readmission requiring an intervention in elderly patients in this study.

Mortality within 30 days after CRS and HIPEC in Sweden is low, with a rate of only 0.2%. This is lower than the 30-day mortality rate (7.7%) presented by Ihemelandu et al. [34]. Moreover, the in-hospital mortality rate is only 1%, which is at the lower end of mortality rates (0.9 to 5.8%) reported by several high-volume HIPEC centres [35, 36].

The coding of comorbidity data has become a more recent phenomenon in Sweden, as it is now partly being used for healthcare reimbursement. However, earlier in our study period, this was not the case, and therefore, reliable comorbidity data is not available to adjust the risk ratios in this study. Nonetheless, most patients being considered for this treatment in general did not have extensive comorbidities, and whilst this is a definite limitation, the authors do not believe it changes the risk factors as identified in this study.

## Conclusion

In-hospital morbidity appears similar to previous studies, and the postoperative mortality rate was low at 1%. However, there is a significant number of readmissions occurring with almost half of the postoperative interventions during the first 6 months occurring outside the HIPEC centre setting.

It is clear that a number of patients experience late complications leading to reoperations at the referral hospital outside the HIPEC centre may not be known to the HIPEC surgeon. Hence, more organized collaboration between referring hospitals and HIPEC centres is desirable. Moreover, our results confirmed that gastric resection and advanced age are two important predictors of morbidity in CRS and HIPEC.

## Data Availability

The dataset that supports the findings of this study is available from the corresponding author upon a reasonable request. The data are not publicly available due to ethical restrictions.

## Abbreviations

CI: Confidence interval
CRS: Cytoreductive surgery
et al.: And others
HIPEC: Hyperthermic intraperitoneal chemotherapy
i.e.: For example
ICD: International Statistical Classification of Diseases and Related Health Problems
Inc.: Incorporation
OR: Odds ratio
PM: Peritoneal metastases
